# Research on Claw Motion Characteristics and Cavitation Bubbles of Snapping Shrimp

**DOI:** 10.1155/2020/6585729

**Published:** 2020-09-20

**Authors:** Yuliang Yang, Shimu Qin, Changchun Di, Junqi Qin, Dalin Wu, Jianxin Zhao

**Affiliations:** ^1^Shijiazhuang Campus, Army Engineering University, Hebei 050003, China; ^2^China Aerodynamics Research and Development Center, Mianyang Sichuan 621000, China; ^3^Unit 63961 of PLA, Beijing 100020, China

## Abstract

Snapping shrimp produces a high-speed jet through the rapid closure of the snapper claw, which stimulates the formation of cavitation bubbles of various shapes. In order to explore the fast motion characteristics of snapper claw, the formation and change process of cavitation, and the physical principles underlying the biological phenomena, the equivalent model of snapper claw was constructed through CT scanning technology. A high-speed camera was used to capture the claw's motion characteristics, thereby simulating the production of cavitation bubbles by snapping shrimp. The results show that the rotation speeds of different species of snapping shrimps are different, as well as their motion characteristics. Cavitation is formed by the interaction of the pressure drop caused by the vortex at the nozzle with the inertia of the liquid inside the socket. Under the influence of the jet, the shapes of bubbles change from ring to cone, and eventually collapse into bubble clouds.

## 1. Introduction

Snapping shrimp is a member of the *Alpheidae* family. It has asymmetrical claws, the larger of which can grow to about half its body size [1]. The claw has a protruding plunger (pl) on the dactyl (d) and a matching socket (s) on the immobile propus (p) [2]. During predation, the snapping shrimp rapidly closes the larger claw [3], draining the water into the socket and forming a high-speed water jet of 32 m/s [4]. Among them, the speed of the water jet is related to the sex of the shrimp and the size of the claw [5]. Because the high-speed water jet injection causes cavitation due to the strong pressure reduction effect of water and the rupture of the cavitation bubbles in front of the claw, the snapping shrimp generates a loud cracking noise of up to 210 dB at the source [6]. The shock wave released by the collapse of cavitation bubbles is about 80 kPa at a distance of 4 cm [1], which is enough to stun and even kill the prey. When the claw is closed [7], there is an angular offset between the dactyl and the propus, which prevents physical contact between these parts, indicating that the noise is caused by the bubble collapse.

The snapper claws exhibit extremely high closing velocities, with a measured value of 3500 rad/s [8]. The rapid closure mechanism is closely related to the structure of the claw. The cocking pivot joints and cocking slip joints evolve on the claw between the dactyl and the propus [9, 10], which facilitates the rapid closure of the claw. The cone-shaped micropapillae units on the dactyl also have the effect of reducing underwater resistance, which facilitates the rapid closure motion of the snapper claw [11].

The cavitation generated by snapping shrimp is a kind of vortex cavitation [12]. The original vortex with a cavitation ring is located in front of the claw. The volume of the bubble is related to the closure velocity [13]. The reason why the high-speed jet is generated is not because the axial momentum is large, but the momentum is converted into the maximum strength of the leading vortex. However, these findings are based on a simplified nozzle model, which can differ from the mechanism used by real shrimp. The collapsed cavitation produces high pressures and temperatures [4], thereby effectively forming a plasma with photons and shock waves through energy focusing [14]. In order to avoid the destruction of the claw structure by high-energy movement, it was found that the materials of the claw have good temperature resistance [15] and are subjected to more intense contact stresses [7].

The previous investigations mainly focused on the mechanical properties, surface morphology, snapping sound, and evolution of the snapper claw. However, the studies about the mechanism of cavitation generated by snapping shrimps and the characteristics of snapper claw motion are still blank. In order to elucidate the biophysical characteristics of these small shrimps, this paper studied the structure of snapper claw through CT scanning and established a 3D model of snapper claw, which was then used to simulate the formation of cavitation bubbles. A high-speed camera was applied to capture and record the entire process of snapper claw rapid closure and the formation and development process of cavitation bubbles to analyze its motion characteristics and cavitation mechanism. Based on the combination of the above, the simulation results and the images captured by the high-speed camera can provide insight into the generation and development of cavitation bubbles of the snapper claw.

## 2. Materials and Methods

### 2.1. Preparation of Snapping Shrimp

In this experiment, samples were collected from the coastal area of Fujian, China, in July 2018. A total of 6 adult male snapping shrimps that had molted for over one week were selected for the experiment. According to the category of snapping shrimps, the species and sizes of the shrimps used in the experiment are as follows: Sample No.1 (*Alpheus macroskeles*, 44 mm with body length, and 8 mm with snapper claw size), Sample No.2 (*Alpheus brevicristatus*, 50 mm with body length, and 10 mm with snapper claw size), Sample No.3 (*Alpheus brevicristatus*, 62 mm with body length, and 9 mm with snapper claw size), Sample No.4 (*Alpheus brevicristatus*, 48 mm with body length, and 8 mm with snapper claw size), Sample No.5 (*Alpheus acutocarinatus*, 68 mm with body length, and 12 mm with snapper claw size), Sample No.6 (*Alpheus acutocarinatus*, 74 mm with body length, and 11 mm with snapper claw size). The snapping shrimps were housed in seawater (temperature: 25 ± 1°C, salinity: 1.024) and were fed frozen shrimp every three days. Before the experiment, the snapping shrimps were numbered with small labels.

### 2.2. Measurement of Snapper Claw Motion Characteristics

The use of high-speed cameras (V2512, Phantom) ensures that the movement of the snapper claw and the formation and development of cavitation bubbles can be accurately captured and recorded. The experimental system consists of a 50∗25∗30 cm aquarium, a vibration-isolated platform, and two high-speed cameras. Snapping shrimp was fixed on the vibration-isolated platform by covering its carapace and claw with plasticene. The positions of the claw and cameras need to be adjusted to the center of the camera's field of view. Through the stimulation of a soft brush, the claw made a raising and rapid closure of the dactyl, and then the high-speed cameras were triggered to shoot. The cameras located above and vertical to the animals were both set to 99000 fps, 10 *μ*s exposure time, and 512∗512 pixels. After the experiment, the calibration plate with a resolution of 2 mm was placed on the plane perpendicular to the claw of the camera to provide a reference for calculating the size, distance, and angle during image postprocessing.

The actual size of the image was determined based on the pixel coordinates of the picture and the length of the calibration plate. The length of 4 mm on the calibration plate corresponds to the average value of 56 pixels, so the length of each pixel is 71.43 *μ*m. The contour of the snapper claw was obtained according to the gray value of the image through MATLAB, and the angles between the dactyl and the propus were determined. Through curve fitting, the relationship between the angle and the time was obtained. Moreover, the relationship between the angular velocity and the angular acceleration was also obtained through the derivative with time.

Because the motion characteristics of No.1 shrimp (*Alpheus macroskeles*) was better than other samples, cavitation research was carried out by carrying out CT scanning and CFD simulation on it.

### 2.3. Computed Tomography of the Snapper Claw

The snapper claw was removed with scissors, and then cleaned with an ultrasonic cleaner (KQ-200KDE, Kunshan ultrasonic instruments CO., Ltd.) for 30 minutes to remove salt residues, and then frozen in a vacuum freeze-drying machine (FD-1C-50, Beijing Bo Kang laboratory instruments CO., Ltd.). The dried snapper claw was tightly fixed with tape, and then observed on a CT machine (nanoVoxel-3000, Tianjin Sanying Precision Instruments CO., Ltd) with a scanning accuracy of 6.52 *μ*m.

### 2.4. Computational Fluid Dynamics Simulation

**Figure 1:**
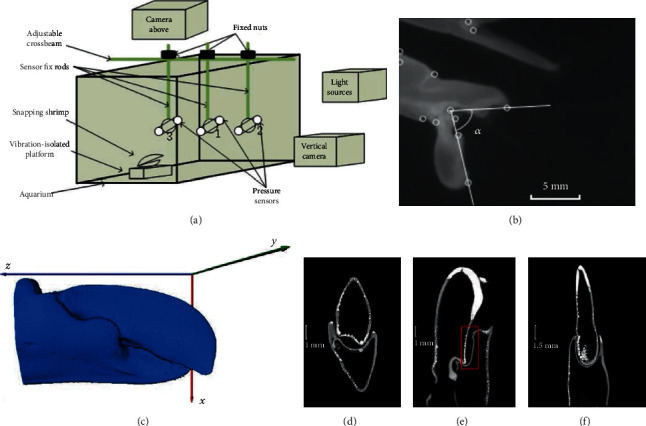
(a) Schematic view of the experimental device. The vibration-isolated platform is placed in the center of the aquarium, and the sensors are fixed at a distance of 3 cm in front of the snapper claw. (b) Gray value image of the snapper claw from the side. The inflection points of the claw are marked by circles, and the opening angle is measured to be 82.4°. (c) 3D reconstructed model of snapper claw with a scale of 1 : 1. (d) CT slices of shrimp claw in the x-y direction. The dactyl is inserted into the socket to form a cavity closed on both sides. (e) CT slices of shrimp claw in the x-z direction. The red rectangle indicates the cavity and nozzle structure. (f) CT slices of shrimp claw in the y-z direction. The dactyl is tightly surrounded by socket, representing a fine hermetic seal.

The stack of CT slices was imported into the visualization software (Avizo 8.1) and then the claw is rendered in 3D visualization. The reconstructed 3D surface data of the claw was then exported to the CAD software (UG NX10). The main functional geometries of the claw were separated into the moving part (dactyl), the stationary part (propus), and the connection part to the pivot axis. According to the measurement results of high-speed photography, the angle of the claw was set to 82.4°, as shown in Figure 1(b).

Since the jet and the cavitation bubble generated by the snapping shrimp were positioned close to the snapper claw, in order to optimize the calculation accuracy and time, the CFD simulation mesh model containing internal and external parts was established, in which tetrahedral meshes with the size of 30 *μ*m were used in the internal part, and hexahedron meshes with the size of 1 mm were used in the external part. The mesh size expansion ratio between the internal and external parts was 1.2 : 1. After verification of grid independence, the number of cells met the calculation requirements.

The Reynolds number redefined the ratio of inertial forces to viscous forces in the fluid [16], which can determine the flow type.

(1) Re=ρvdμ,

where *v* is the jet velocity, *ρ* is the fluid density, *μ* is the viscosity, and *d* is the characteristic size of the claw nozzle. With the typical values of *v* ≈ 32 − 70 m/s, *ρ* = 1024 kg/m^3^, *μ* = 1.003*e* − 03 kg/(m∗s), *d* ≈ 0.2 mm, the Reynolds number is about 6534-14293, indicating that the type of jet produced by snapping shrimp is turbulence.

The FLUENT software package was used for the CFD simulation. In the simulation, the Navier-Stokes equations were used to simulate a Newtonian fluid whilst maintaining mass, momentum, and energy. In addition, the achievable k-e turbulent model and VOF model were selected. The application of the PISO method was to determine the iterative solutions of the pressure field and velocity field. The selected solvers and predefined parameters in the simulation are listed in Table 1.

A dynamic mesh was used to simulate the movement of the dactyl, which is essential to accurately simulate the process. According to the high-speed photography results, the closure velocity of the claw was accelerated, from the initial velocity of 70 rad/s to the final velocity of 4392 rad/s, and the total time was only about 885 *μ*s. Subsequently, the claw motion information was written to a profile to command the dactyl movement. For dynamic mesh, the *smoothing* and *remeshing* mesh methods were selected. The settings of the dynamic mesh are listed in Table 2.

## 3. Results

### 3.1. Movement Characteristics of the Snapper Claw

**Figure 2:**
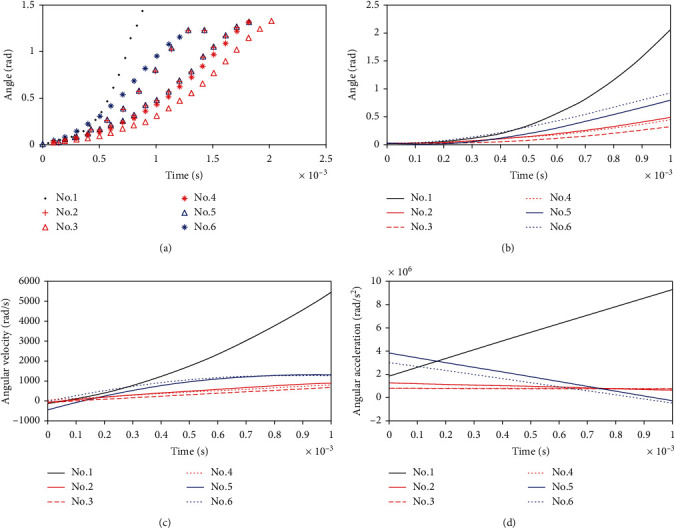
Motion information of the claw. (a) Curve of claw angle and time. (b) Angle of claw as a function of time. (c) Angular velocity of claw as a function of time. (d) Angular acceleration of claw as a function of time.

The high-speed camera accurately observed the movement of the snapper claw. In response to the stimulation, the snapping shrimp tilted its dactyl to the maximum angle and stayed in that position for about a second, then the dactyl moved to close the claw at an extreme rapid velocity around the joint. The curve of the snapper claw motion angle is shown in Figure 2(a). The 3^rd^ order polynomial can fit the relationship between the angle of the claw motion and time well, as shown in Figure 2(b). The parameters of the curve fitting (*f*(*x*) = *p*1∗*x*^3^ + *p*2∗*x*^2^ + *p*3∗*x* + *p*4) were shown in Table 3.

Taking the derivative of the angle gave the angular velocity, which is an acceleration process from less than 100 rad/s to more than 1000 rad/s finally, as shown in Figure 2(c). Using the same method, the angular acceleration was derived as follows. The No.1 shrimp (*Alpheus macroskeles*) had the highest angular velocity of about 4500 rad/s, and the angular acceleration continued to increase to nearly 10^7^ rad/s^2^. The maximum angular velocity of the No. 2-4 shrimps (*Alpheus brevicristatus*) was about 1000 rad/s, and the angular acceleration almost remained unchanged at 10^6^ rad/s^2^. The maximum angular velocity of the No. 5 and No. 6 shrimps (*Alpheus acutocarinatus*) was about 1400 rad/s, and the angular acceleration continued to decrease, from the initial 4 × 10^6^ rad/s^2^ to 0 or even negative value.

### 3.2. Characterization of Claw Structure

The images presented in Figures 1(c) and 1(d) show the 3D reconstructed model and the slices of the snapper claw in different directions. When the claw is closed, a cavity is formed between the dactyl and the propus, as indicated by the red rectangle in Figure 1(e). In the process of closing the claw, the dactyl moves around the axis on the propus. The velocity reaches its maximum value at the moment when the dactyl is completely closed. The plunger on the dactyl provides the initial liquid velocity by replacing the liquid in the socket. Due to the sealing of the socket and the propus, the liquid can only flow out of the socket through the orifice on the side of the claw, resulting in a high-speed jet similar to the function of the nozzle structure.

### 3.3. Formation and Development of Cavitation Bubbles

**Figure 3:**
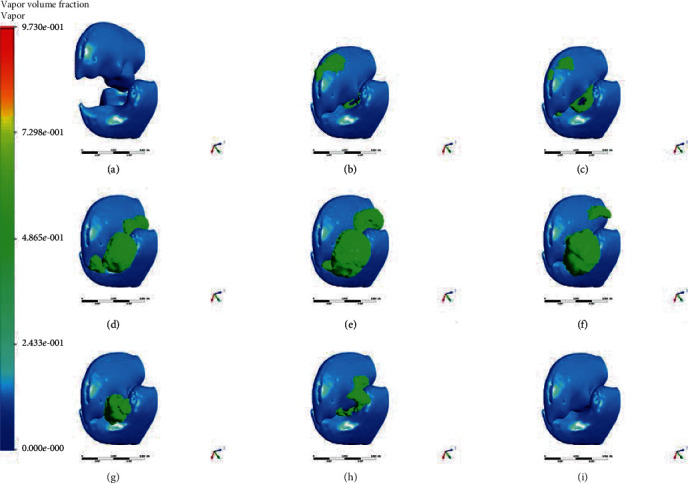
Indicative instances of the snapper claw model closure. The green part represents the cavitation bubble with the vapor volume of 50%.

The simulation results of the snapper claw model are shown in Figure 3. In the simulation and calculation of cavitation bubbles, the isosurface shows bubbles with a volume fraction of 50% (the green part in the figure). When the claw is closed from the maximum angle (Figure 3(a)), the angular velocity gradually increases. With the movement of the claw, bubbles are first formed in the corners of the socket (Figure 3(b)). When the claw is about to close, a toroidal bubble appears around the orifice formed by the dactyl and propus (Figure 3(c)). When the claw motion stops, the bubble continues to grow and moves in the direction of the jet (Figure 3(d) and (e)). Afterwards, under the influence of the ambient pressure, the bubble begins to shrink and eventually collapses (Figure 3(f)–(h)) and disappears (Figure 3(i)).

## 4. Discussion

### 4.1. Analysis of the Movement Characteristics of the Snapper Claw

According to the law of conservation of energy, the larger the volume, the more water is contained in the socket, so more kinetic energy is required to drive the liquid. In addition, the larger the volume of the claw, the greater the area of contact with water during movement, so the greater the resistance. Taking into account the above two reasons, the movement characteristics of different types of snapping shrimps in the experiment are different. Among the samples, the smallest snapping shrimp, *Alpheus macroskeles* (No. 1), had a tiny snapper claw that was only about 8 mm long, so the resistance it received when moving in the water was small, and the energy provided by its muscle contraction was enough to squeeze out the fluid in the socket cavity. Therefore, both the angular velocity and acceleration increased when the shrimp closed its claw. The *Alpheus brevicristatus* (No. 2-4) had larger snapper claws than those of the No.1 shrimp, so the resistance they suffered when moving in the water was also greater, resulting in a slower closure velocity. However, due to the small volume of the socket cavity of this type of shrimp, its water storage capacity was small, and the resistance of the liquid to the dactyl was low during squeezing. Combining two points, the closure angular acceleration of the claw of this type of shrimp did not change much. Among the test samples, the *Alpheus acutocarinatus* (No.5-6) had the largest volume with an average dactyl length of 12 mm. Due to their slender and flat contour of the dactyls, which can reduce resistance, the closure velocity of snapper claw was faster than the second kind of shrimps, about 1400 rad/s. On the other hand, due to the large volume of the socket cavity, higher energy was required to squeeze out the stored liquid, resulting in insufficient energy provided by muscle contraction to overcome the resistance of the liquid, so the angular acceleration of the claw motion continued to decrease.

### 4.2. Cavitation Inception

**Figure 4:**
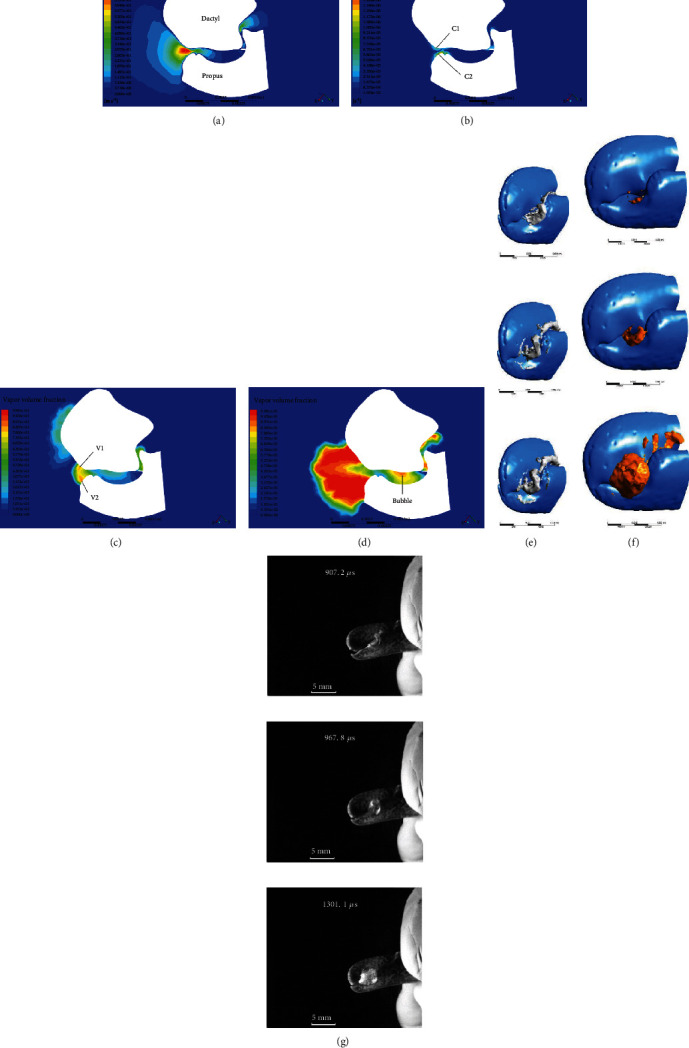
(a) The contour of jet velocity. The core velocity is much higher than the surroundings, forming a submerged jet in water. (b) The contour of vortex. Vortices are formed around the orifice of the nozzle, causing the pressure drop. (c) The contour of vapor. At the position of the vortex, cavitation incepts and the initial shape is toroidal. (d) The cavitation bubble in the socket cavity due to inertia. (e) Vortex core regions are indicated in grey. It gradually develops into a ring structure close to the dactyl. (f) The development of the cavitation bubble. The bubble is formed first at the nozzle orifice, and then changes from a toroidal bubble to a more conical one. (g) The pictures of cavitation bubbles generated by snapper claw.

The contour of the cavitation inception of the snapper claw is shown in Figure 4. The velocity of the jet generated by the claw flow through the nozzle is about 70 m/s in the center (Figure 4(a)), which is much faster than the velocity of the surrounding flow. Under the squeezing of the plunger, the liquid in the socket cavity is ejected, so that the jet kinetic energy increases inside the cavity. When the liquid reaches the nozzle, the jet will continue to move along the wall of the claw. Under the influence of the inverse pressure gradient (dp/dx > 0), the jet velocity near the wall gradually decreases. At this time, the boundary layer begins to increase in thickness, and separates from the nozzle to form a vortex, and finally merges into the mainstream. As shown in Figure 4(b), the vortex structures C1 and C2 are first formed around the nozzle, and the directions of the two vortices are opposite, with the vortex of about 300,000. According to Bernoulli's law, the pressure in the region with high velocity can be decreased and the presence of the vortex causes a pressure drop. The pressure at the core of the vortex is the lowest, accompanied by cavitation inception. Cavitation bubbles V1 and V2 appear around the nozzle and are connected to the dactyl and the propus, as shown in Figure 4(c). The induced liquid depressurization caused by the vortex can be expressed as [17]:

(2) $$\begin{array}{c} p_{R}-p_{C}=\frac{\rho R^{2}\Omega^{2}}{2}, \end{array}$$

where *p*_*R*_ is the pressure at the vortex radius, and *p*_*C*_ is the pressure at the vortex core. The liquid density *ρ* is 1024 kg/m^3^, and the vortex radius *R* is about 0.15 mm. The vorticity is twice the instantaneous principal axis angular velocity of the strain-rate tensor of the fluid element, so the angular velocity *Ω* is about 150000 rad/s, with an average pressure drop of 259200 Pa (about 2.5 atm). Since the pressure at the vortex core is sufficiently lower than the saturated vapor pressure of 3540 Pa, this pressure difference between the two can lead to cavitation.

When the dactyl stops moving, the socket is not filled by the plunger, and the liquid in the cavity continues to flow outward along the nozzle due to inertia, resulting in cavitation inside the cavity, as shown in Figure 4(d). The formula for calculating the pressure drop in the channel is [18]:

(3) $$\begin{array}{c} \Delta p=\rho \frac{l}{S}\frac{d\phi }{dt}, \end{array}$$

where *l* and *S* are the length and the cross-sectional area of the channel, and *ϕ* is the flow rate.

After the bubble in the cavity leaves the socket, it enters the low-pressure area of the nozzle and then merges with the toroidal bubble generated by the vortex. For snapping shrimp, these two sources of cavitation work together to form the bubble.

### 4.3. Development of Vortex Cavitation

After cavitation starts at the nozzle, the vortex moves with the flow of the jet.  Figure 4(e) shows the contour of the vortex at the nozzle when the claw is closed, where the grey parts represent the vortex regions. The vortex first appears around the nozzle orifice, and then develops into a ring structure close to the dactyl in a similar manner to the air vortex cannon [19]. The core velocity of the jet produced by the claw is about 70 m/s initially, and then gradually decreases. The vortex ring structure is formed around the jet. As the jet develops, its radius gradually increases. According to the jet theory, under the influence of the viscosity of the liquid, the boundary layers of the jet continuously fall off, resulting in the vortex filling of the area of the jet boundary. The vortex will cause turbulence, which will entrain any fluid in the static state into the jet. With the development of turbulence, the entrained fluid increases, and the boundary of the jet gradually expands to both sides. The flow rate *q* increases along the path [20].

(4) $$\begin{array}{c} \frac{q}{q_{0}}\propto \sqrt{\frac{x}{d}}, \end{array}$$

where *q*_0_ is the flow at the nozzle, and *d* is the feature size of the nozzle. Therefore, the vortices form a large vortex ring structure around the jet, and its size continues to increase.

The bubble morphology is simulated and shown in Figure 4(f), in which the orange part represents the contour of 20% vapor volume fraction. The bubble is ejected from the nozzle on the side of the claw, and there is a certain angle between the moving direction of the bubble and the claw. The initial cavitation bubble appears as a ring structure, which continuously expands and stretches to a conical structure in contour along the direction of the jet under the impact of a high-speed jet. Sadovskii et al. [21] studied the morphological changes in cavitation bubbles produced by high-speed submerged jets. At the moment of jet generation, a cavity appeared as a narrow hollow torus in the plane perpendicular to the jet direction. The transverse diameter of the cavity was much larger than the jet diameter. When the jet was ejected from the nozzle, the cavity surface evolved into an approximately cylindrical surface that grew in the direction of jet motion. After the jet was generated, the closure of the bottom surface of the cavity resulted in the formation of an axial jet inside the cavity. The ever-evolving reentrant jet traversed the cavity along its axis of symmetry to form a new conical cavity, which had a convex surface as it penetrated into the liquid. The bottom surface of the cavity where the jet was generated advanced in the direction of the jet. Therefore, the cavitation bubbles generated by the snapping shrimp evolved from the initial ring-shaped bubbles into more conical bubbles and moved in the direction of the jet. A high-speed camera was used to photograph the process of cavitation bubbles produced by the claw of snapping shrimp. As can be seen from Figure 4(g), after the claw was closed, the toroidal bubble overflowed from the side of the claw and continuously expands in the cone structure. In addition, the direction of its motion is at an angle relative to the claw. Therefore, the high-speed imaging confirms the validity of the theoretical and simulation results. It can be concluded that there are two main functions of the jet. One is to transfer the axial momentum of the jet to the vortex. The enhanced vortex forms a low-pressure zone, which advances with the vortex, causing continuous expansion of cavitation bubbles. The other is to guide the movement of the vortex ring and cavitation bubbles.

### 4.4. Collapse of Cavitation Bubbles

**Figure 5:**
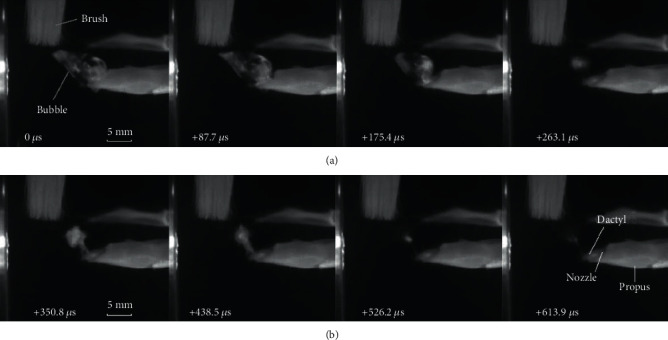
High-speed images from the top of the claw. (a) The pictures of the compression of cavitation bubbles. The volume of the conical bubble reduces to is the minimum. (b) The pictures of the rebound of cavitation bubbles. The bubbles rebound many times without a specific shape and eventually disappear.

There are two stages of the bubble collapse, namely, the compression stage and the rebound stage [22]. During the compression stage, due to the decrease in vortex intensity, the pressure outside the bubble is greater than the internal pressure, causing the bubble to start compressing. At this stage, the bubbles are relatively stable and the disturbance increases slowly. There is a highly unstable nonspherical disturbance in the rebound stage, which is manifested by the fierce breakup of bubbles and the formation of many small bubbles. Under the continuous rupture and rebound, the bubbles form a visible cloud. Through the high-speed camera, the collapse process of the bubbles generated by snapping shrimp is recorded. Through careful observation, it can be found that the nonspherical bubbles begin to shrink after expanding to the maximum volume. During the shrinkage process, the outline of the bubble remains clear and the shape remains unchanged, indicating that it is in the compression stage, as shown in Figure 5(a). After the bubble shrinks to its minimum volume, it begins to rebound and expand, changing from a single conical bubble to many cloud bubbles. These bubbles burst and rebound continuously, and then the cloud disappears gradually. This process is the rebound stage, as shown in Figure 5(b).

The Rayleigh-Plesset equation is widely used to describe the collapse of a bubble [23].

(5) $$\begin{array}{c} \frac{p_{g}-p_{\infty }}{\rho }=R\frac{d^{2}R}{{dt}^{2}}+\frac{3}{2}{\left(\frac{dR}{dt}\right)}^{2}+\frac{4\nu }{R}\frac{dR}{dt}+\frac{2S}{\rho R}, \end{array}$$

where *R* is the radius of the bubble, *ρ* is the density of the liquid, *ν* is the dynamic viscosity, *S* is the surface tension, *p*_*g*_ and *p*_∞_ are the liquid pressure at the interface and far away from the bubble, respectively.

**Figure 6:**
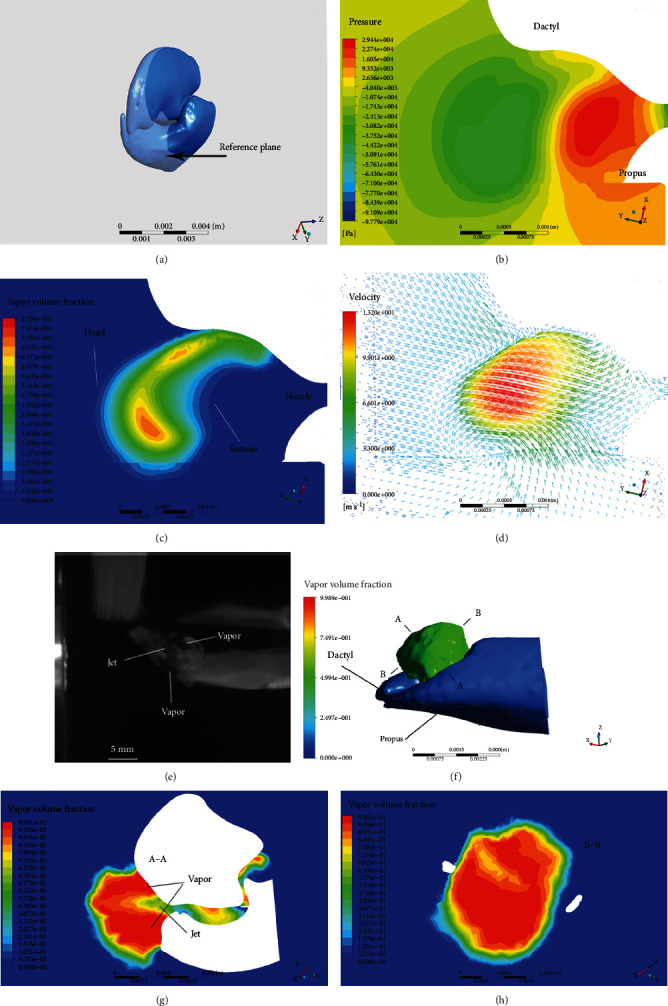
(a) Reference plane for the simulation results, showing the flow field of the snapper claw along the nozzle. (b) Contour of the pressure on the claw side when the bubble collapses. The pressure on both sides of the bubble is different, and the pressure on the bottom is higher. (c) When it is about to burst, the bubble is compressed into a concave shape, pointing to the head of the bubble. (d) Speed vector of the flow field. When the bubble bursts, the fluid on both sides moves toward the middle of the bubble, and the jet velocity from the bottom of the bubble pointing to the head is greater. (e) Picture of the cavitation bubble in the maximum state. The nonspherical bubble includes the jet in the middle and vapor around. (f) Simulation result of cavitation bubble with vapor volume fraction of 50%. (g) Contour of vapor volume fraction along the axis of the bubble. (h) Contour of vapor volume fraction along the radial cross-section of the bubble.

According to the R-P equation, it can be inferred that the collapse process of the nonspherical bubble is related to the local curvature radius of the initial shape of the bubble [24]. According to the simulation results, when the bubble bursts, the forces on both ends of the bubble are different. The pressure in the head area is lower than the pressure in the tail area, which causes the bubble to collapse from the bottom to the head, and generates a micro jet directed to the head, as shown in Figures 6(a).

Therefore, it is estimated that the cavitation bubbles generated by the shrimps are directional. When the snapping shrimp is hunting, its claw aims at the prey, and the shock wave and the microjet generated by the collapse of the bubble both act on the target, which has little effect on the snapping shrimp itself.

### 4.5. Structure Characteristics of Cavitation Bubbles

Figure 6(e) shows the maximum volume of cavitation bubbles produced by the claw. It can be seen from the outline that the cavitation bubble is conical, in which the bottom surface is elliptical and the front surface is elongated. The main body of the bubble is transparent, and there is an opaque jet inside. In the simulation results shown in Figure 6(f), along the axis of the bubble (A-A), the inside of the bubble was not all water vapor, but there was a conical cavity structure as shown in Figure 6(g). The bubble is not the initial ring structure, but a symmetrical nonspherical shape. The dimensionless parameter *σ* was applied to describe the cavitation.

(6) $$\begin{array}{c} \sigma =\frac{p_{\infty }-p_{v}}{1/2\rho V_{\infty }^{2}}, \end{array}$$

where *p*_∞_ is the absolute pressure, and *p*_*v*_ represents the vapor pressure of the liquid. *V*_∞_ is the jet velocity, and *ρ* is the liquid density. The cavitation number describes the possibility that the fluid cavitates or not when the high-speed flow reaches such low pressure. Where *p*_∞_ = 0.103 MPa, *V*_∞_ = 32 ~ 70 m/s, *p*_*v*_ = 3540 Pa, and *ρ* = 1024 kg/m^3^. Therefore, the cavitation number of bubbles generated by the snapping shrimp is *σ* = 0.04 ~ 0.19. When the cavitation number is less than or equal to 0.2, the submerged jet will produce asymmetric cavitation, accompanied by the axial reentrant flow [25], which is consistent with the principle of cavitation bubbles generated by the snapper claw.

As shown in Figure 6(h), along the radial cross-section of the bubble (B-B), the vapor volume fraction presents an annular distribution with a lower center, and liquid is the main component. The vapor volume fraction in the middle is relatively higher, with dominant vapor. When external bubbles come into contact with water, the vapor volume fraction gradually decreases.

## 5. Conclusions

This paper reviewed the research status of snapping shrimps and analyzed the movement characteristics of several kinds of snapping shrimps and the dynamic changes of cavitation bubbles, which provide a useful reference for biomimetic research on the movement mechanism and cavitation mechanism of snapping shrimp.

This study investigated the motion characteristics and the cavitation mechanism of the snapper claw, as well as the development and structure of cavitation bubbles. Cavitation bubbles are generated by the snapping shrimp through the rapid closure of the snapper claw. This is an acceleration process with velocity ranging from 1000-4500 rad/s, which is relevant to the structure of the snapper claw. The average pressure drop at the claw nozzle is about 2.5 atm, which is sufficient to cause cavitation. The cavitation bubble is based on both Bernoulli's law and inertia. When the cavitation number of the bubbles generated by the snapping shrimp is less than 0.2, an asymmetric cavitation vortex is generated. Under the influence of the flow jet, the cavitation bubble deforms from the initial toroidal bubble to a conical one. When it is about to burst, the bubble is compressed into a concave shape, accompanied by a micro jet directed toward the head of the bubble.

## Figures and Tables

**Table 1:** Parameters of fluent simulation.

Parameters	Predefined value			
Solver	Pressure-based		Transient	
Solution methods	PISO		PRESTO, second order upwind	
Multiphase flow model	VOF		Realizable *k* − *ε*, Standard Wall function	
Cavitation model	Zwart-Gerber-Belamri		3540 pa	
Materials	Primary phase	Water-liquid	Second phase	Water-vapor
	Density	1024 kg/m^3^	Density	0.02558 kg/m^3^
	Viscosity	1.003*e* − 03 kg/(m∗s)	Viscosity	1.26*e* − 06 kg/(m∗s)

**Table 2:** Settings of the dynamic mesh.

Smoothing method	Parameters	Remeshing method	Parameters
Spring constant factor	0.01	Minimum length scale (*m*)	0
Convergence tolerance	0.001	Maximum length scale (*m*)	0
Number of iterations	20	Maximum cell skewness	0.7
Laplace node relaxation	1	Size remeshing interval	1

**Table 3:** Curve fitting parameters of angle with time.

Sample	*p*1	*p*2	*p*3	*p*4	*R* ^2^
1	1.24*E*+09	9.25*E*+05	-136.1	0.026	0.9962
2	-1.13*E*+08	6.53*E*+05	-78.79	0.02616	0.9973
3	-9.46*E*+06	3.97*E*+05	-98.84	0.0232	0.9976
4	-2.23*E*+06	3.91*E*+05	27.61	0.01768	0.999
5	-6.82*E*+08	1.91*E*+06	-454.4	0.02831	0.9926
6	-5.83*E*+08	1.50*E*+06	-13.16	0.01602	0.9991

## Data Availability

The data used to support the findings of this study are available from the corresponding author upon request.
